# Synthesis of Silane Functionalized LDH-Modified Nanopowders to Improve Compatibility and Enhance Corrosion Protection for Epoxy Coatings

**DOI:** 10.3390/molecules29040819

**Published:** 2024-02-10

**Authors:** Alireza Aminifazl, Darshan Jayasinghe Karunarathne, Teresa D. Golden

**Affiliations:** Department of Chemistry, University of North Texas, 1155 Union Circle #305070, Denton, TX 76203, USA; alirezaaminifazl@my.unt.edu (A.A.);

**Keywords:** Zn-Al LDH, decavanadate, silane coupling agent, epoxy coating, corrosion protection

## Abstract

Novel modified Zn-Al LDH/epoxy coatings are synthesized and applied to steel substrates, providing active corrosion protection and improved barrier properties. This protective coating is made by combining Epon 828 as a polymer matrix with modified layered-double-hydroxy (LDH) nanoparticles acting as corrosion inhibitor containers. To synthesize the coatings, nitrate was intercalated into Zn-Al-LDH layers through an aqueous co-precipitation method to obtain Zn-Al LDH-NO_3_, and decavanadate replaced nitrate within the LDH layers through an anion exchange process to obtain Zn-Al LDH-(V_10_O_28_)^6−^. The intercalated LDH was functionalized by silanization with (3-aminopropyl)triethoxysilane (APTES) to increase the compatibility of the LDH inhibitor nanocontainers with epoxy resin and produce a protective coating. To protect the mild steel substrate, functionalized LDH nanopowders were dispersed into the epoxy resin, mixed with a polyamide hardener (Epikure 3571), and applied and cured to the metal surface. Surface morphology, structure, and chemical composition were determined for the modified LDH nanopowders using scanning electron microscopy, energy-dispersive X-ray analysis, X-ray diffraction, infrared spectroscopy, X-ray photoelectron spectroscopy, and thermogravimetric analysis. Corrosion protection of the coating system was studied using long-term immersion testing and potentiodynamic polarization studies in a 3.5 wt.% NaCl solution.

## 1. Introduction

Metals and their alloys are used in many different industries, including construction, marine, automotive, oil and gas, and transportation, due to their high strength and ductility [1,2]. However, a major problem encountered for steel materials is corrosion when exposed to harsh environmental conditions. For steel and iron alloys, corrosion can occur under such conditions as high humidity, exposure to salts, and extreme pH and temperature. This metal corrosion is a worldwide problem and results in large costs for many industries [1,2]. The corrosion of metals costs more than USD 4 trillion annually, which is ~3% of the nation’s gross domestic product [1]. According to one estimation, by using any available corrosion control tool or practice, the cost of corrosion can be reduced by 15–35% [3].

Currently, the design and development of corrosion-protective coatings for metals is of great interest [1]. Galvanization, surface phosphating, the use of chromium compounds, and cathodic protection are some methods to prevent steel corrosion. However, the disadvantages associated with these methods include environmental hazards, labor costs, poor adhesion, and weak corrosion performance, leading to the development of other protective coatings. Recently, researchers have focused on formulating organic coatings using a variety of techniques and additives, such as surface modification and inhibitive fillers, to enhance the barrier and provide active corrosion protection [4,5,6,7,8], while still retaining the desired properties such as mechanical strength and optical appearance. Organic coatings are used mostly as a barrier when there is no defect already present. Epoxy resin-based organic coatings are widely used to shield steel against extreme corrosion [9,10,11,12]. To actively guard against corrosion, these coatings can serve as both a barrier to prevent the transfer of corrosive species to the substrate and as a coating loaded with corrosion inhibitors. These epoxy coatings can exhibit strong adhesion, excellent durability, good mechanical properties, and high chemical resistance and work as a defensive barrier against corrosion and metal oxidation [13]. The main disadvantage of epoxy resin coatings is their porosity and weak performance when exposed to light. The pores in the surface allow for the penetration of aggressive chemicals into the metallic substrate, resulting in corrosion. Based on the literature, the porosity of epoxy coatings can be minimized by using inorganic fillers [11].

Layered double hydroxides (LDHs) are a class of anionic clays that are synthetic lamellar compounds [14,15,16] having a general formula of MII_1−x_MIII_x_(OH)_2_(An^−^)_x_/n·mH_2_O, where MII are divalent metal cations such as Co, Ni, or Zn; MIII are trivalent cations such as Al or Fe; and An^−^ are anions such as CO_3_^2−^ or NO_3_^−^. Positively charged stacking layers of LDHs are neutralized by anions, which are located between the interlayers. These hydroxylated layers can be separated by interlayered anions and water molecules [14,15,16]. Their unique anion exchange property and rich chemistry make them a promising inorganic filler for polymer composites and coatings [17,18]. LDHs can be reservoirs for inhibitive anions, which are released when exposed to corrosive environments. This anion-exchange ability of LDH can improve corrosive inhibition by two main functions: (1) trap corrosive anions (i.e., chloride ions) and (2) release corrosion inhibitive ions [19,20,21]. Phosphates, molybdates, tungstates, vanadates, nitrites, and borates have been used as inorganic inhibitors. Benzotriazole (BTA), mercaptobenzothiazole (MBT), imidazoline, 8-hydroxyquinoline (8-HQ), and aliphatic amines have been incorporated into the LDH interlayers as organic corrosion inhibitors [22]. When these loaded LDHs are added to a coating, the coating will display inhibitive effects and improve corrosion protection. LDH can be synthesized by a co-precipitation process in aqueous media, microwave synthesis, ultrasound methods, hydrothermal treatments, electrochemically, or sol-gel methods [23,24,25].

To add these modified LDH nanoparticles as inorganic fillers to epoxy-type coatings, a chemical agent is needed to enhance the compatibility and miscibility of the inorganic filler with the organic epoxy. Trialkoxy silanes are commonly used as coupling agents. Three hydrolyzable groups mediate the formation of silanol groups for bonding to mineral surfaces, and one organofunctional group is used for reactivity with resin to enhance compatibility and improve chemical resistance. These compounds are also considered adhesion promoters because they make strong bonds between the resin and mineral substrates [26,27]. There are limited works on the functionalization of LDHs in polymer composites [28,29,30]. In the current study, 3-aminopropyltriethoxy silane (APTES) was used to functionalize modified LDHs and incorporate the LDH into an epoxy matrix. This improved compatibility and increased the corrosion protection of the coating. Inorganic modified LDHs with decavanadate have unique properties that make them attractive for use in polymer composites, including high thermal stability, high surface area, and good chemical reactivity. Additionally, the functionalization of these LDHs can further enhance their properties, such as improving dispersion in the polymer matrix and increasing interfacial adhesion. Decavanadate LDHs have not been extensively studied in this context. This work provides new insights into the potential of these materials as corrosion inhibitors and expands the range of available materials for use in anti-corrosion coatings. Finally, this work demonstrates the successful synthesis and incorporation of silane-functionalized decavanadate LDHs in epoxy coatings to improve the corrosion protection properties of the coatings. This has practical applications in industries where metal substrates are exposed to harsh environments, such as the oil and gas industry or marine coatings.

## 2. Results

### 2.1. Production and Characterization of Inorganic Fillers

**Figure 1:**
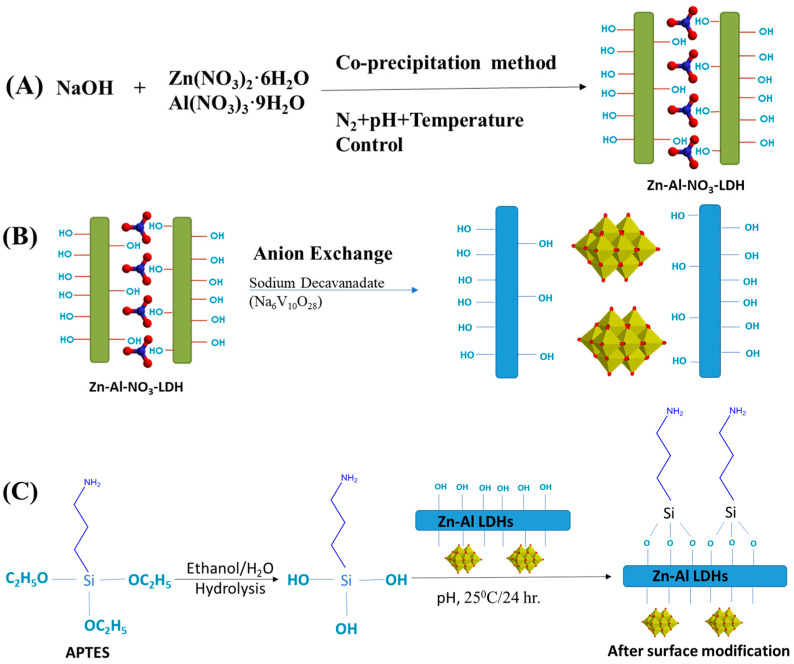
Schematic representation of (**A**) synthesis of Zn-Al-LDH-NO_3_ (LDH-N) powders; (**B**) anion exchange process to modify LDH nanopowders with decavanadate anions (LDH-D); and (**C**) surface functionalization of the nanoparticle fillers with silane coupling agents (LDH-NF and LDH-DF).

The schematic representation of the synthesis processes to produce the inorganic fillers for the coatings (as detailed in the experimental section) is shown in Figure 1. The synthesis of the Zn-Al-LDH-NO_3_ (LDH-N) powders was carried out through a co-precipitation route under a nitrogen atmosphere to avoid contamination of the LDH with carbonate anions (Figure 1A). The anion exchange reaction to produce the Zn-Al LDH-decavanadate (LDH-D) inorganic filler is shown in Figure 1B. Both of these inorganic nanoparticle fillers were further functionalized via a silanization process using (3-aminopropyl)triethoxysilane (APTES) to make the LDH nanoparticles (Figure 1C) compatible for later addition in the epoxy coating. These functionalized nanoparticles are referred to as LDH-NF and LDH-DF. The characterization of LDH-N, LDH-D, LDH-NF, and LDH-DF nanopowders was carried out and described in the next sections before addition to epoxy for the final coating process.

#### 2.1.1. FT-IR Analysis of LDH-Modified Powders

**Figure 2:**
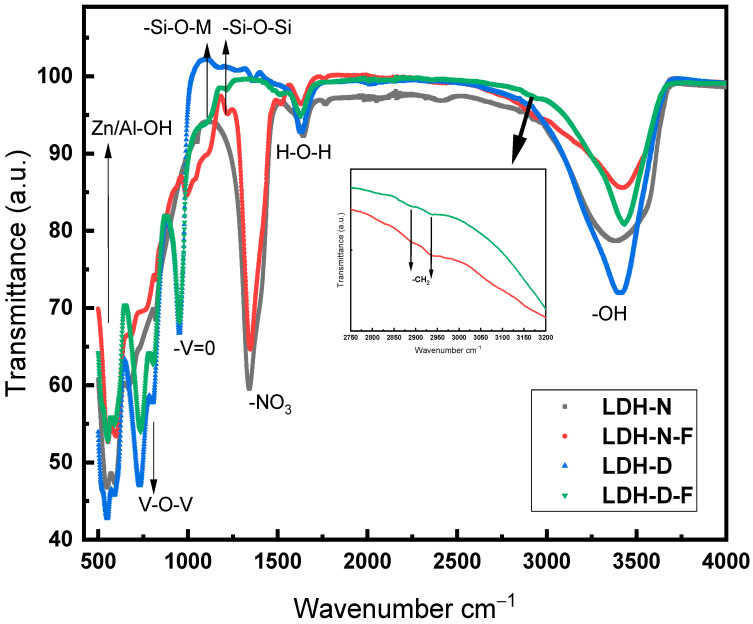
FT-IR spectra of LDH-N, LDH-NF, LDH-D, and LDH-DF synthesized nanopowders.

Fourier transform infrared (FT-IR) spectroscopy was run in the frequency range of 400–4000 cm^−1^ to study nitrate Zn-Al LDH (LDH-N), silane functionalized nitrate Zn-Al LDH (LDH-NF), decavanadate Zn-Al LDH (LDH-D), and silane functionalized decavanadate Zn-Al LDH (LDH-DF) synthesized nanopowders. The FT-IR spectra (Figure 2) of all samples showed broad asymmetric and symmetric stretching vibrations in the region of 3400–3700 cm^−1^, corresponding to the Zn-OH and Al-OH layers as well as the -OH groups of the interlayered H_2_O molecules. There is also a weak IR band at 1635 cm^−1^, which is attributed to the deformation and bending vibration of H_2_O. The sharp peak appearing at 1350 cm^−1^, which is related to the N-O bonds in nitrate, demonstrates the presence of NO_3_^−^ in the interlayer spacing for LDH-N and LDH-NF and disappears when decavanadate replaces nitrate in the anion exchange process for LDH-D and LDH-DF 31-37]. This shows successful intercalation of the decavanadate group between LDH platelets. The stretching and bending vibrational modes of Al–O–Al, Zn–O–H, and metal-oxygen-metal bonds in LDH structures are seen in the range of 400–800 cm^−1^. There is no nitrate anion peak for the LDH-D and LDH-DF samples due to the anion exchange reaction, and instead there is an intense peak at 950 cm^−1^, which belongs to the symmetric stretching mode of terminal -V=O groups, and another peak at 670 cm^−1^, attributed to the antisymmetric and symmetric stretching modes of V-O-V chains in decavanadate [31,32,33,34,35,36,37]. The stretching bands between 2851 and 2921 cm^−1^ (insert) are for the C-H groups in the functionalized samples [38]. A table listing the FT-IR assignments for the LDH-N, LDH-NF, LDH-D, and LDH-DF and supporting citations indicating successful nitrate and decavanadate anion intercalation for the synthesized powders is in the supplementary section as Table S1. In addition, the specific region in the range of 1050–1250 cm^−1^ has two distinct peaks corresponding to the Si-O stretching vibrations, which are shown in Supplementary Data as Figure S1. These peaks confirm the surface functionalization with APTES on the surface for the LDH-NF and LDH-DF samples.

#### 2.1.2. Thermogravimetric Analysis (TGA)

**Figure 3:**
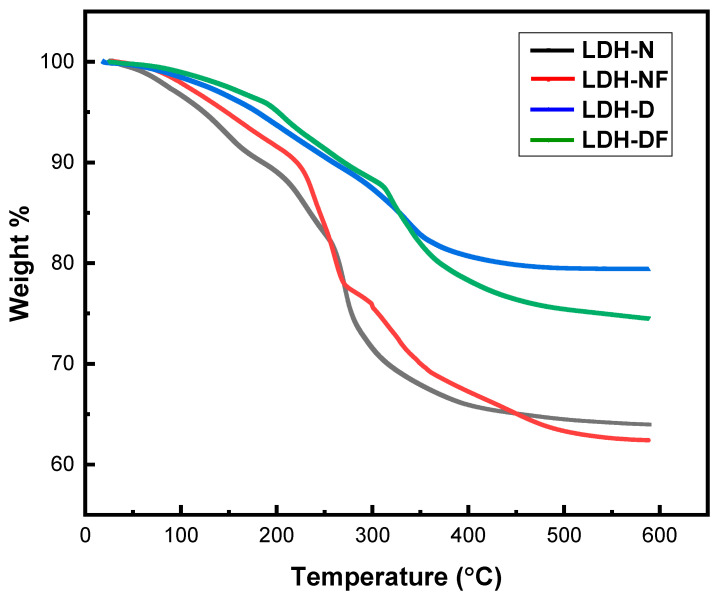
TGA profiles of the synthesized LDH-N, LDH-NF, LDH-D, and LDH-DF nanopowders.

The TGA graphs of the four different powder samples are shown in Figure 3. For the TGA runs (Figure 3), they all exhibited similar weight loss curves but at different percentages. For each sample, there were three distinct regions. The first region is related to the removal of intramolecular and interlayer water and takes place in a temperature range from ~25 °C to 150 °C. The second region, from ~150 °C to 300 °C, is the partial elimination of structural hydroxyl groups in the brucite layer (dihydroxylation). The last and third regions are between ~300 °C and 600 °C and belong to the total dehydroxylation of the host layers and the decomposition of intercalated anions [37]. Additionally, for the functionalized samples, grafted organic silane breakdown occurs in the third phase at temperatures ranging from 300 to 600 °C. The % weight loss for these steps is calculated for the LDH nanopowders and listed in Table 1. There is a total weight loss equal to 36.1, 37.6, 21.7, and 25.4% for LDH-N, LDH-NF, LDH-D, and LDH-DF, respectively. LDH-NF and LDH-DF recorded weight losses of 13.4 and 13.7%, respectively, in the third step, compared to LDH-N and LDH-D, which recorded losses of 8.6 and 7.6%. This is due to the degradation of organic groups that were grafted onto the surface of LDHs during the grafting process utilizing APTES. This outcome provided additional proof that the APTES graft on the LDHs was successful. Also, the total weight loss for the decavanadate-intercalated LDH (LDH-D and LDH-DF) is less than for the nitrate samples. This can be seen for the calculated decomposition values (T_d_) given in Table 1, where the nitrate anion containing samples have Td values around 262–275 °C and the decavanadate anion containing samples have Td values around 324–340 °C, about a 65 °C difference. While the nitrate anions are lost through thermal decomposition, the polyoxovanadate anions do not decompose in the gaseous phase at the temperature ranges we used, thus giving a more thermally stable filler for the coatings [25,31,33,35,37].

#### 2.1.3. XRD Analysis

**Figure 4:**
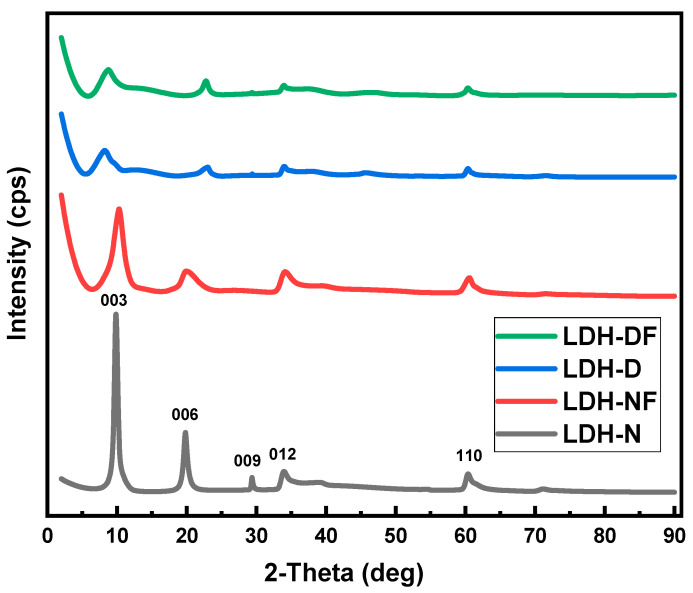
XRD patterns of LDH-N, LDH-NF, LDH-D, and LDH-DF synthesized nanopowders.

XRD patterns were also recorded for the LDH samples (Figure 4). The main characteristic diffraction peaks for the LDH structure were observed and indexed as (003), (006), (009), (012), and (110) (PDF card 00-055-0193). The diffraction peaks observed for LDH-N at 2θ of ~10 and 20° correspond to the (003) and (006) reflections, and the (110) reflection is observed at 61.2°. Compared with the LDH-N pattern, the LDH-D and LDH-DF (003) and (006) reflections are shifted to lower 2θ values. This shift indicates a larger layer spacing for the intercalation process, where decavanadate anions have replaced the smaller nitrate ions, thus increasing basal spacing. The (003) d-spacing values for the LDH samples increase from 8.98 to 11.27 Ǻ for LDH-N and LDH-D, respectively [31,32,33,34,35,38]. The d-spacings for the LDH-N to LDH-NF samples are the same as for the LDH-D to LDH-DF samples. This indicates that the d-spacing values are due to the intercalation of the anions only and are not affected when the LDH-N and LDH-D are functionalized with APTES [38].

#### 2.1.4. Morphology and Composition of Synthesized LDH Powder Nanoparticles

**Figure 5:**
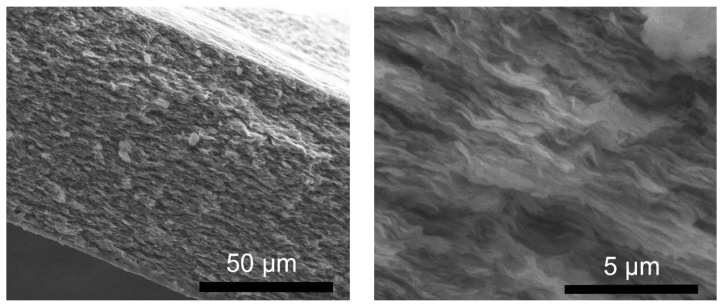
SEM cross-sectional micrograph of LDH-NO_3_ nanopowder dried on an aluminum foil surface at different magnifications.

**Figure 6:**
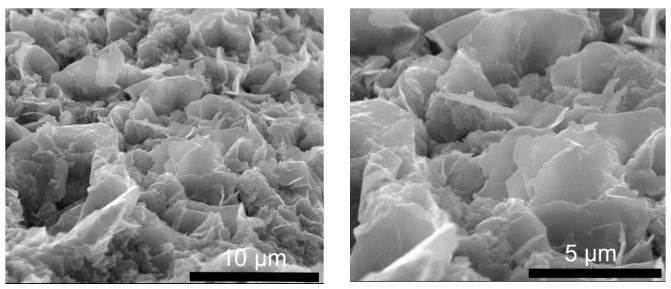
SEM top-view micrograph of LDH-NO_3_ nanopowder particles dried on an aluminum foil surface at different magnifications.

**Figure 7:**
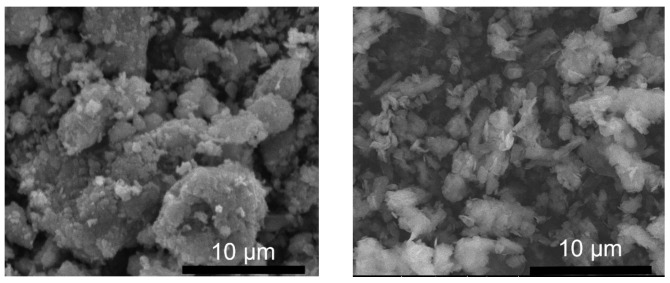
SEM micrograph of LDH-NF (**left**) and LDH-DF (**right**) ground nanoparticles.

Scanning electron microscopy (SEM) was used to study the morphologies of the synthesized LDH powders. Figure 5 shows the cross-sectional view of the LDH-N sample dried on an Al surface. The sample has a layered morphology consisting of stacked plate-like particles. This is a typical morphology for many LDH-type materials [39]. The width of these plates is in the range of a few nanometers, and the length is a few micrometers, as seen from the top view for the LDH-N (Figure 6). After functionalization, SEM images of the LDH-NF and LDH-DF ground samples showed agglomeration at different sizes up to a few micrometers (Figure 7). Compared to the non-functionalized powders, the morphology of these LDHs has changed due to the subsequent synthesis and functionalization operations.

Energy dispersive X-ray (EDX) analysis was run for all the powders to verify the synthesis results and support FT-IR data (Figure S2 in the Supplementary Section). Analysis of all samples indicated they contained Zn, Al, and O. Also, the ratio of zinc to aluminum is approximately 2:1 in all the samples, consistent with the LDH structure [40]. According to the EDS results for LDH-N, the only elements present are Zn, Al, O, and N, indicating the formation of a Zn-Al LDH gallery with nitrate ions intercalated between the layers. For the synthesized LDH-NF, there are N peaks as well as Si and C peaks, indicating successful silane functionalization with APTES. The successful anion exchange reaction replacing nitrate ions with decavanadate ions can be seen for the LDH-D powders. The EDS spectra show the appearance of the V peak and the disappearance of N in the spectrum. The characteristic nitrogen peak vanishes as a result of the replacement of nitrate anions by decavanadate anions. Like LDH-NF, surface functionalization on LDH-D is supported by distinctive peaks in the EDS spectra that contain Si and C.

#### 2.1.5. X-ray Photoelectron Spectroscopy of Synthesized LDH Powder Nanoparticles

**Figure 8:**
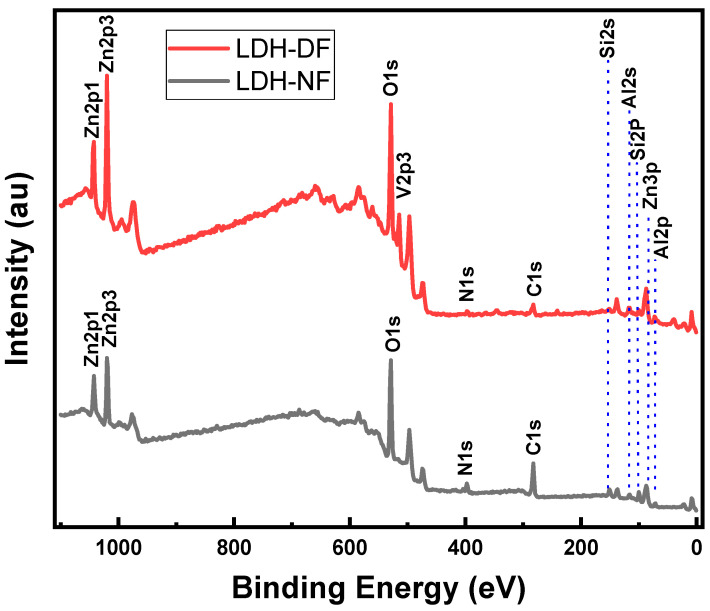
XPS survey spectra of LDH-NF and LDH-DF samples.

**Figure 9:**
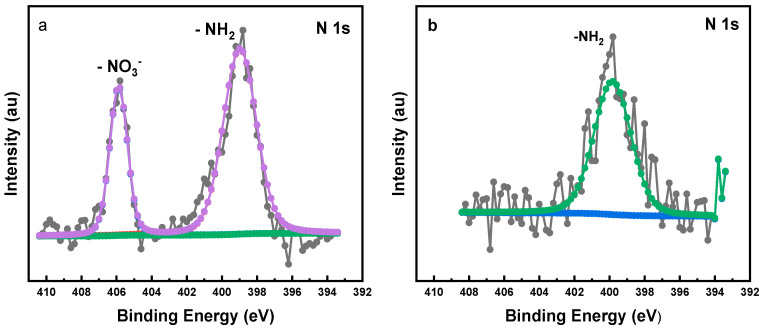
XPS N 1s spectrum for (**a**) LDH-NF (raw data—black, background-green, fit line-purple) and (**b**) LDH-DF samples (raw data—black, background—blue, fit line—green).

Also, X-ray photoelectron spectroscopy (XPS) of the samples confirms the successful anion exchange and functionalization of the nanopowders. The XPS survey spectra for LDH-NF and LDH-DF are shown in Figure 8. Peaks corresponding to the elements Zn, Al, Si, N, C, and O are present for LDH-NF, and the same elements plus V are present for LDH-DF, indicating successful intercalation and functionalization of the nanopowders. For the anion exchange reactions (Figure 9), the N 1s spectrum of the LDH-NF sample has two components, one at 405.8 eV and the other at 399.9 eV, which are attributed to the -NO_3_ interlayer anions and the -NH_2_ group on APTES, respectively. In contrast to LDH-NF, the LDH-DF N 1s spectrum only exhibits one component at 399.8 eV, which belongs to the amine group on APTES, and offers another proof of the replacement of nitrate with vanadate anions. Also, there is a shift in the O 1s binding energy from 531.7 eV for LDH-NF to 530.6 eV for LDH-DF, which is most likely due to an increase in the electron density on the latter caused by the effective electronic interaction between the decavanadate anions and the Zn-Al-double hydroxide layers (see supplementary data Figures S3 and S4) [41]. The plots for Al 2p, Zn 2p, O 1s, Si 2p, C 1s, and V 2p components of the XPS spectrum can be found in the supplementary data (Figures S3 and S4). Table 2 lists the XPS assignments for the LDH-NF and LDH-DF nanopowders. Note that LDH-NF and LDH-DF have Zn 2p, Al 2p, Si 2p, and C 1s binding energies at very close values [41,42].

### 2.2. Production and Characterization of Epoxy-LDH Coatings

After the successful production of the LDH-functionalized nanopowders, these were added to an epoxy matrix to produce uniform coatings. Functionalization of the nanopowders with APTES also changes the hydrophobic properties of the samples. The decavanadate-intercalated LDH (LDH-D) has a relatively lower contact angle (29°) since the water droplet tends to spread out and form a flat shape on the surface, indicating more hydrophilic behavior. However, the silane-functionalized LDH (LDH-DF) has a much higher contact angle (90°), signifying a more hydrophobic surface (supplementary data in Figure S5). Higher hydrophobic properties can help repel water and increase corrosion resistance.

**Figure 10:**
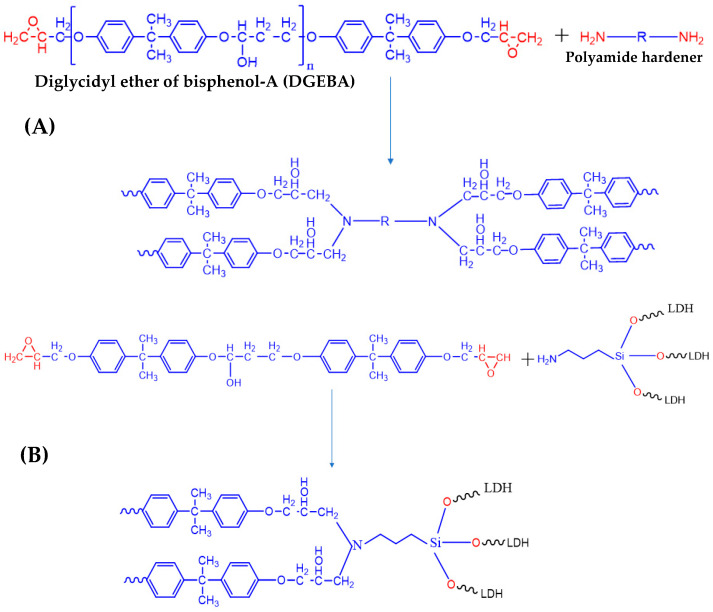
(**A**) Epoxy resin and amide hardener ring opening reaction. (**B**) Functionalized LDH (APTES) powders added to the reaction scheme in (**A**) react with the resin.

**Figure 11:**
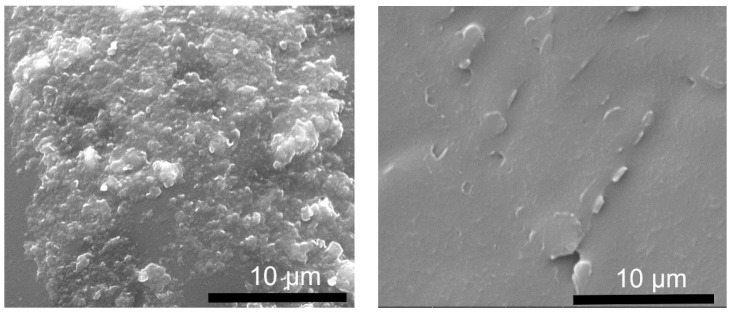
SEM micrographs of the LDH-DF epoxy coating surface (**left**) and cross-section (**right**).

Figure 10A shows the reaction scheme to produce the epoxy coatings from the resin and amide hardener, in which the nucleophilic attack of the amine group on the electrophilic carbon in the epoxy ring causes the ring to open and a covalent bond to be formed between amine compounds and the epoxy resin. This curing reaction of resin and hardener leads to the formation of a crosslinked polymer network that imparts the final hardened epoxy with its desired physical and chemical-resistant properties. In this reaction, the functionalized LDH powders can also react with the resin, as shown in Figure 10B. Approximately 3% of the functionalized LDH powder is added to the reaction, leaving an excess of resin to react with the hardener in order to produce the final coating. The bond between decavanadate-intercalated LDH and the resin matrix (supplementary data in Figure S6) is strengthened by combining a silane coupling agent with an epoxy resin. Unbound NH_2_ groups on functionalized LDH can interact with the weak oxirane rings in the epoxy resin, resulting in exceptional compatibility between the LDH and the epoxy matrix. This connection creates a direct link between the inorganic and organic phases, consequently maximizing interfacial adhesion and leading to better dispersion and higher compatibility of functionalized LDHs in the epoxy matrix. Figure 11 shows the SEM micrographs of the top and cross-section of the final coatings. High-speed mixing and sonication can help achieve a good dispersion of modified LDH nanopowders in an epoxy matrix, which can have a major impact on the samples’ protection performance. The coatings were smooth and compact, with good dispersion of nanopowders within the matrix. The corrosion resistance properties of these coatings were tested using several techniques.

#### 2.2.1. Corrosion Studies of Epoxy/LDH Coating: Salt Solution Immersion Test

**Figure 12:**
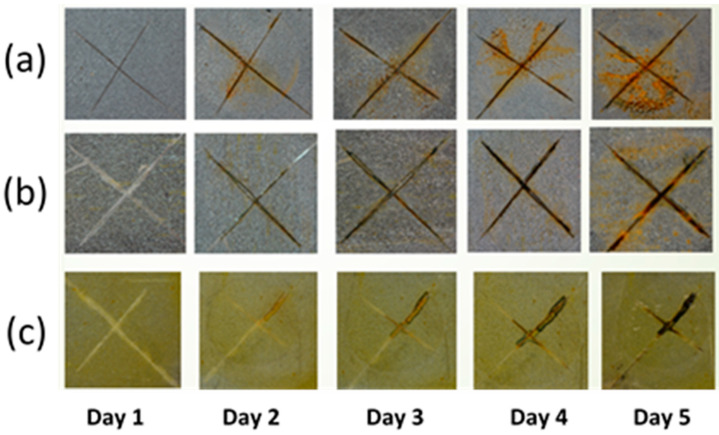
Salt solution immersion in 3.5 wt% NaCl for (**a**) bare epoxy, (**b**) LDH-NF epoxy, and (**c**) LDH-DF epoxy coatings on steel for days 1–5.

The anticorrosion capabilities were studied for the final epoxy coatings using several techniques. The bare epoxy, LDH-NF epoxy, and LDH-DF epoxy samples were cut using a blade to form an X pattern and immersed in a 3.5% salt solution for 1, 2, 3, 4, and 5 days. Their visual appearance and results are displayed in Figure 12. Pure epoxy coatings showed severe corrosion attack along the scratched area and underneath the coating after only 5 days, while the LDH-NF epoxy coating, due to the presence of LDH particles and improved barrier quality, showed slightly less corrosion attack. The best corrosion resistance, however, is seen with the LDH-DF epoxy coatings, where the nanopowders contain decavanadate. When the corrosive molecules and ions, such as water and chloride, penetrate through the polymer coating, electrochemical corrosion takes place and pitting occurs on the steel substrate [20,21,22,43,44]. During immersion in a salt solution, LDH-DF particles exhibit inhibition properties in two different ways to protect the steel structure. First, plate-like nanoparticles hinder the passage of aggressive ions via a passive barrier protection mechanism [45]. Second, as active protection, LDH particles can release decavanadate and zinc ions to reduce the contents of the corrosive ions and can trap aggressive chloride anions through the anion exchange process. Other researchers have shown good results with different intercalated Zn-Al films trapping chloride ions [46,47].

#### 2.2.2. Corrosion Studies of Epoxy/LDH Coating: Potentiodynamic Polarization

**Figure 13:**
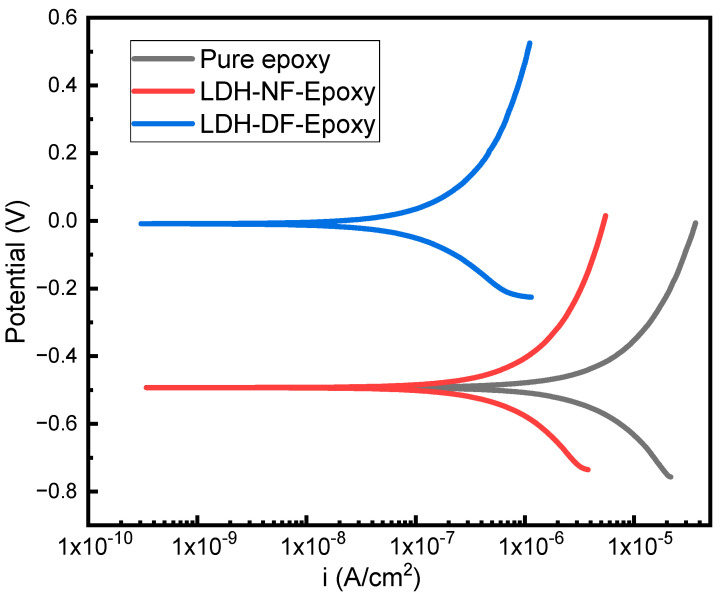
Potentiodynamic polarization plots for pure epoxy, LDH-NF epoxy, and LDH-DF epoxy after immersion in a 3.5 wt% NaCl solution for 30 days.

The corrosion resistance for the LDH-NF and LDH-DF epoxy-coated steel is determined using potentiodynamic polarization (Figure 13) after immersion in a 3.5 wt% NaCl solution for 30 days. The potentiodynamic data for the samples shows that the corrosion current density (i_corr_) decreased when the steel surface was coated by LDH-incorporated epoxy coatings, and the corrosion potential (E_corr_) increased (positive shift) for the LDH-DF epoxy coating. The corrosion current, i_corr_, decreased by 77 and 96% when the steel substrate was coated with LDH-NF and LDH-DF epoxy, respectively. E_corr_ had a positive (cathodic) shift from −0.49 V for bare epoxy coating to −0.006 V for LDH-DF epoxy coating. Polarization resistance (R_p_), which represents the coating resistance against current flow and corrosion, increased to 5.7 × 10^4^ and 3.8 × 10^5^ Ω/cm^2^ for LDH-NF and LDH-DF epoxy coatings, respectively, compared to the bare epoxy sample, indicating enhanced corrosion protection. Table 3 lists all the fitted parameters as follows: corrosion potential (E_corr_), corrosion current density (i_corr_), anodic slope (β_a_), cathodic slope (β_c_), and polarization resistance (R_p_) for the samples run with potentiodynamic polarization. All measured corrosion parameters indicate the best corrosion resistance is for the LDH-DF epoxy coating on the steel substrate.

## 3. Materials and Methods

### 3.1. Materials

Zn (NO_3_)_2_·7H_2_O, Al (NO_3_)_3_·9H_2_O, NaOH, HNO_3_, NaCl, and NaVO_3_ were purchased from Fisher Scientific (Waltham, MA, USA) and Aldrich (St. Louis, MO, USA). All were of analytical grade and used as received, without further purification or processing. Mild steel substrates were purchased from a local supplier (Denton, TX, USA). Industrial-grade epoxy resin (Epon 828), polyamide hardener (Epikure 3571), and aminopropyltriethoxysilane (APTES) as silane coupling agents were acquired from Hexion (Columbus, OH, USA) and ShinEtsu (Tokyo, Japan), respectively. In-house deionized and decarbonated water was used in all the experimental processes.

### 3.2. Synthesis of Nitrate and Decavanadate Intercalated LDHs

The synthesis of Zn-Al-LDH-NO_3_ was carried out through a co-precipitation route under a nitrogen atmosphere to avoid contamination of the LDH with carbonate anions. Zn (NO_3_)_2_·6H_2_O (0.02 M) and Al (NO_3_)_3_·9H_2_O (0.01 M) were prepared in 700 mL of decarbonated water, and then concentrated NaOH was added slowly to the solution under vigorous stirring at room temperature [31,32,33]. The solution was heated to 95 °C and kept there for 24 h. The resulting residue was recovered by centrifugation at a speed of 3500 rpm for 6 min, washed 3 times with decarbonated water and ethanol, and then dried at 90 °C for 12 h. In the second step, Zn-Al-decavanadate−LDH was obtained through an anion-exchange process using Zn-Al-NO_3_−LDH as a base material [34,38,48]. Moreover, 1 g of LDH-NO_3_ was added to 100 mL of decarbonated water and stirred for 1 h at room temperature. The pH of the solution was adjusted to 4.5 by adding 2 M HNO_3_. Next, 1.0 g of NaVO_3_ was dissolved in 50 mL of decarbonated water, and then 2 M HNO_3_ was slowly added in order to reach a pH of 4.5. The resulting orange-colored solution confirmed changing VO_3_^−^ to decavanadate anions (V_10_O_28_^6−^). The orange decavanadate solution was then added to the LDH-NO_3_ suspension and stirred for 4 h at room temperature. A yellow solid product was centrifuged out and washed repeatedly with decarbonated water and ethanol, then dried at 70 °C for 12 h and ground into powder.

### 3.3. Preparation of Silane Functionalized Decavanadate and Nitrate LDHs

The decavanadate-intercalated LDH was first ground into a fine powder and then added to an ethanol solution (1 g to 40 mL) in a two-necked flask at 50 °C and stirred for 1 h. Moreover, 1 mL of (3-aminopropyl)triethoxysilane (APTES) was added to a 90% mixture of ethanol/water (9 mL:1 mL). Furthermore, the prepared APTES solution was added dropwise to the decavanadate LDH suspension, stirred at high speed, and kept for 24 h under a nitrogen atmosphere. The final product was silane-functionalized decavanadate LDHs, which dropped out of the solution as a yellow precipitate. This product was washed with ethanol and centrifuged three times to remove any unreacted silane coupling agent, then dried at 50 °C for 24 h. The same procedure was carried out to functionalize NO_3_ LDHs to obtain silane-modified NO_3_ LDHs for comparison.

### 3.4. Preparation of Epoxy/LDH–APTES Decavanadate Composite

The polymeric solution method was used for the preparation of epoxy/LDH decavanadate composites, so that silane-decavanadate-LDH particles (3 wt.%) were thoroughly dispersed into diluted epoxy resin (15 mL) (Epon 828) and mixed with a mechanical stirrer at 1400 rpm at room temperature. Stoichiometric content of polyamide hardener (Epikure 3571) (resin-to-hardener ratio of 65:35) was added to the epoxy system and completely mixed, then the resin was applied using a film applicator on mild steel coupons and left for 48 h to fully cure and dry before corrosion testing. For the curing reaction of epoxy resin and hardener, one reactive amine group of APTES results in three-dimensional crosslinking and a strong bond with epoxy functional groups, respectively, which enhances adhesion, creates good dispersion, and has high compatibility for LDH-functionalized nanoparticles in epoxy [35,36].

### 3.5. Characterization of Modified LDH Powders and Epoxy-Functionalized Coatings

After the synthesis of intercalated LDHs and silane-modified LDH powders, various techniques were used to characterize the synthesized powders and identify their structure, chemical composition, and morphology before and after addition to the epoxy coating system.

A Perkin–Elmer Spectrum Two FT-IR spectrometer was used for analysis to monitor functional groups during the synthesis process. Each sample was scanned 16x’s in a range from 4000–400 cm^−1^. The sample was placed on an ATR stage for analysis.

Thermogravimetric analysis (TGA) was run to determine the thermal stability of the nanoparticles. A TA Instrument SDT650 was used to study the effect of inorganic modifier addition to the LDH in the samples. A temperature ramp of 20°/min was used, starting at 25 °C and running up to 600 °C, with changes monitored over time.

X-ray diffraction (XRD) analysis was used to study the layered structure, basal spacing, and exfoliation of the synthesized LDH and modified LDH. A Rigaku Ultima III XRD was used with a Cu Kα X-ray tube (λ = 0.1541 nm) and set at 40 kV and 24 mA. The scans were run from 5–100° 2θ at 5°/min speed. The Bragg equation was used to calculate the basal spacings for the LDHs [49].

A FEI Quanta 200 environmental scanning electron microscope (ESEM) with an X-ray dispersive spectroscopy (EDS) attachment was used to study morphological and elemental changes in the samples. SEM was conducted at an accelerating voltage of 15 kV with a spot size of 3.0 nm and a 10 mm working distance using a secondary electron (SE) detector. Images taken by the SE detector showed the morphology of the modified LDHs and final epoxy coatings. Energy Dispersive X-ray Analysis (SEM-EDX) was run to characterize the elemental composition of the LDH particles using a 7.0 nm spot size.

X-ray photoelectron spectroscopy (XPS) was run on a PHI 5000 Versa probe X-ray photoelectron spectrometer with a monochromatic 1486.6 eV Al K radiation source. The samples were prepared by compressing the nanopowders in the form of a freestanding pellet. At a pass energy of 187 eV, the survey scan was acquired for each sample, and then core-level spectra and elemental region measurements were taken at a pass energy of 23.5 eV. To remove any adventitious carbon contamination, the sample underwent ion sputtering, and the charge neutralization system was also used during XPS measurements. Peaks were deconvoluted using CASA XPS software, and charge correction was performed using C 1s (284.8 eV) as the standard.

The hydrophobic properties of the resulting functionalized nanoparticles were investigated by taking pressed pellets of nanoparticle powders and measuring the contact angle. A YSC Technologies HD802 angled viewing microscope (Fremont, CA, USA) and ImageJ software (ver. 1.54f) were used to measure the contact angle formed at the interface between the liquid droplet (water) and the solid surface.

The corrosion behavior of the coatings on the substrate was investigated by long-term immersion in a 3.5% salt solution, and the surface morphology and composition were monitored microscopically. Potentiodynamic polarization (PD) was used to study the corrosion performance of the coated samples. The polarization resistance (R_p_) was obtained from linear polarization resistance (LPR) by scanning the voltage ±20 mV from OCP for all coatings. Poteniodynamic polarization curves were obtained at a scan rate of 0.5 mV/s from +500 to −250 mV. The corrosion current density was calculated using the Stern-Geary equation [50].

## 4. Conclusions

Overall, this work has demonstrated a successful synthesis of silane-modified nitrate and decavanadate-intercalated Zn-Al LDHs and their incorporation into epoxy coatings for corrosion protection. FT-IR and XRD confirmed a complete anion exchange process. As a result of the reactivity of the silane alkoxyl groups with the LDH surface hydroxyl groups and the greater stability of the decavanadate anion compared to nitrate, the heat stability was also increased for the LDH-DF nanoparticles. SEM images confirmed stacking plate-like sheets for LDH-N and showed smaller particles for decavanadate-intercalated LDH. SEM studies also showed a good dispersion of modified LDHs in the epoxy coating on the surface and within the cross-section area. Epoxy coatings without decavanadate anions were also found to be ineffective at protecting mild steel against corrosion when subjected to an immersion test in a salt solution. In contrast, LDH-DF epoxy coatings provide longer and better corrosion inhibition over a 30-day period. Higher corrosion protection performance may be attributable to the release of inhibitor ions and the absorption of aggressive ions, as evidenced by a positive shift in the corrosion potential, E_corr_, and an increase in polarization resistance, R_p_, measured by potentiodynamic polarization for an LDH-DF epoxy coating. Based on these results, it is shown that by modifying inorganic fillers (LDHs) placed within epoxy coatings, the corrosion protection of these coatings can be greatly enhanced. These modifications to the inorganic fillers include the intercalation of a corrosion-inhibiting anion (decavanadate) and the attachment of an organo-silane group to increase the miscibility of the filler within the organic epoxy coating.

## Figures and Tables

**Table 1:** Weight loss% in three different temperature regions, T_10_, and T_d_ calculated for synthesized powder samples from TGA data.

Samples	Weight Loss% (25–150 °C)	Weight Loss% (150–300 °C)	Weight Loss% (300–600 °C)	Total Weight Loss%	T_10_ (°C)	T_d_ (°C)
LDH-N	7.5	21.0	7.6	36.1	184	262
LDH-NF	5.3	18.9	13.4	37.6	220	275
LDH-D	3.5	9.1	8.6	21.7	258	324
LDH-DF	2.6	9.2	13.7	25.4	270	340

**Table 2:** XPS results for the LDH-NF and LDH-DF samples.

Sample	Element	XPS Peak	Chemical Bonding	Peak Values (eV)
LDH-NF	Aluminum	Al 2p	Al-O, Al-OH	74.2
	Zinc	Zn 2p3/2; Zn 2p1/2	Zn (+2)	1021.7; 1044.8
	Nitrogen	N 1s	-NO_3_; NH_2_	405.8; 399.9
	Oxygen	O 1s	Oxides	531.7
	Silicon	Si 2p	Si-O	101.8
	Carbon	C 1s	C-C; C-N	284.5; 285.7
LDH-DF	Aluminum	Al 2p	Al-O, Al-OH	74.0
	Zinc	Zn 2p3/2; Zn 2p1/2	Zn (+2)	1021.8; 1045.9
	Nitrogen	N 1s	-NH_2_	399.8
	Oxygen	O 1s	Oxides	530.6
	Silicon	Si 2p	Si-O	102.2
	Carbon	C 1s	C-C; C-N	284.5; 285.7
	Vanadium	V 2p3/2; V 2p1/2	V-O	516.2; 523.6

**Table 3:** Corrosion values calculated from potentiodynamic polarization data for pure epoxy, LDH-NF epoxy, and LDH-DF epoxy coatings.

Sample	E_corr_ (V)	βa (mV/dec)	βc (mV/dec)	R_p_ (Ω/cm^2^)	I_corr_ (A/cm^2^)
Pure Epoxy	−0.49	676	−397	1.3 × 10^4^	3.4 × 10^−5^
LDH-NF Epoxy	−0.49	767	−333	5.7 × 10^4^	7.7 × 10^−6^
LDH-DF Epoxy	−0.006	855	−249	3.8 × 10^5^	1.2 × 10^−6^

## Data Availability

Data are available upon request from the corresponding author.

## Supplementary Materials

The following supporting information can be downloaded at: https://www.mdpi.com/article/10.3390/molecules29040819/s1. Table S1: FTIR peak assignment for Zn-Al LDH intercalated and functionalized nanopowder samples; Figure S1: FTIR spectra showing Si-O stretching vibrations for the samples; Figure S2: EDS spectra and elemental analysis of LDH-N, LDH-NF, LDH-D, and LDH-DF nanoparticles; Figure S3: (a) Al 2p, (b) Zn 2p, (c) O 1s, (d) Si 2p, (e) C 1s, and (f) N 1s XPS spectra of the LDH-NF sample; Figure S4: (a) Al 2p, (b) Zn 2p, (c) V 2p, (d) O 1s, (e) Si 2p, (f) N 1s, and (g) C 1s XPS spectra of the LDH-DF sample; Figure S5: Contact angle measurements for LDH-D (top) and LDH-DF (bottom) pressed nanopowders; Figure S6: Schematic for the functionalization of decavanadate intercalated LDH (LDH-DF) nanopowders and its interaction with epoxy matrix.
