# Supplementary material for: Synthesis of Silane Functionalized LDH-Modified Nanopowders to Improve Compatibility and Enhance Corrosion Protection for Epoxy Coatings
Source: Molecules. 2024 Feb 10;29(4):819. doi: 10.3390/molecules29040819 (PMC10892364; doi:10.3390/molecules29040819)
Supplement: Supplementary file 1 [file molecules-29-00819-s001.zip › molecules-2773937-supplementary.pdf]

Supplementary Data

Table of Contents

Table S1: FTIR peak assignment for Zn-Al LDH intercalated and functionalized nanopowder samples……….……………………………………………………………..S2

Figure S1: FTIR spectra showing Si-O stretching vibrations for the samples…………….S3

Figure S2: EDS spectra and elemental analysis of LDH-N, LDH-NF, LDH-D, and LDH-DF nanoparticles.…..……………………………………………………..……..……………S4

Figure S3: (a) Al 2p, (b) Zn 2p, (c) O 1s, (d) Si 2p, (e) C 1s, and (f) N 1s XPS spectra of the LDH-NF sample…………………………………………………………………………...S5

Figure S4: (a) Al 2p, (b) Zn 2p, (c) V 2p, (d) O 1s, (e) Si 2p, (f) N 1s, and (g) C 1s XPS spectra of the LDH-DF sample ……….……………………………………………………...S6

Figure S5. Contact angle measurements for LDH-D (top) and LDH-DF (bottom) pressed nanopowders ……………………..……...…………………………………………………....S8

Figure S6: Schematic for the functionalization of decavanadate intercalated LDH (LDH-DF) nanopowders and its interaction with epoxy matrix …………………..……..……….S9

Table S1. FTIR peak assignment for Zn-Al LDH intercalated and functionalized powder samples.

Wavenumber (cm^{-1})	Peak assignment	Reference
3400 (LDH-N, LDH-NF, LDH-D, LDH-DF)	stretching of the OH bond of the hydroxyl groups and H_2O molecules	1, 3
2851–2921 (LDH-NF, LDH-DF)	C–H stretching band	4
1635 (LDH-N, LDH-NF, LDH-D, LDH-DF)	H_2O bending vibration of the interlayer water	1 ,3
1350 (LDH-N, LDH-NF)	$-NO_3$ anti-symmetric stretching mode v_3	1 ,3, 5
980-1120 (LDH-NF, LDH-DF)	Si–O–M stretching vibrations	4
950 (LDH-D, LDH-DF)	-V = O symmetric stretching	4
820 (LDH-N, LDH-NF, LDH-D, LDH-DF)	Al-OH deformation	1 ,3
810 and 732 (LDH-D, LDH-DF)	V-O-V stretching vibrations	2
543 (LDH-N, LDH-NF, LDH-D, LDH-DF)	Zn/Al-OH translation	1 ,3

1. Mahjoubi, F. Z.; Khalidi, A.; Abdennouri, M.; Barka, N. Zn–Al layered double hydroxides intercalated with carbonate, nitrate, chloride and sulphate ions: Synthesis, characterisation and dye removal properties. J. Taibah University for Science 2017, 11, 90-100.

2. Zhou, M.; Yan, L.; Ling, H.; Diao, Y.; Pang, X.; Wang, Y.; Gao, K. Design and fabrication of enhanced corrosion resistance Zn-Al layered double hydroxides films based anion-exchange mechanism on magnesium alloys. Appl. Surf. Sci. 2017, 404, 246-253.

3. Karami, Z.; Jouyandeh, M.; Ali, J. A.; Ganjali, M. R.; Aghazadeh, M.; Paran, S. M. R.; Naderi, G.; Puglia, D.; Saeb, M. R. Epoxy/layered double hydroxide (LDH) nanocomposites: Synthesis, characterization, and Excellent cure feature of nitrate anion intercalated Zn-Al LDH. Progress in Organic Coatings 2019, 136, 105218.

4. Hu, J.; Gan, M.; Ma, L.; Li, Z.; Yan, J.; Zhang, J. Synthesis and anticorrosive properties of polymer–clay nanocomposites via chemical grafting of polyaniline onto Zn-Al layered double hydroxides. Surf. Coatings Technol. 2014, 240, 55-62.

5. Jia, H.; Zhao, Y.; Niu, P.; Lu, N.; Fan, B.; Li, R. Amine-functionalized MgAl LDH nanosheets as efficient solid base catalysts for Knoevenagel condensation. Molecular catalysis 2018, 449, 31-37.

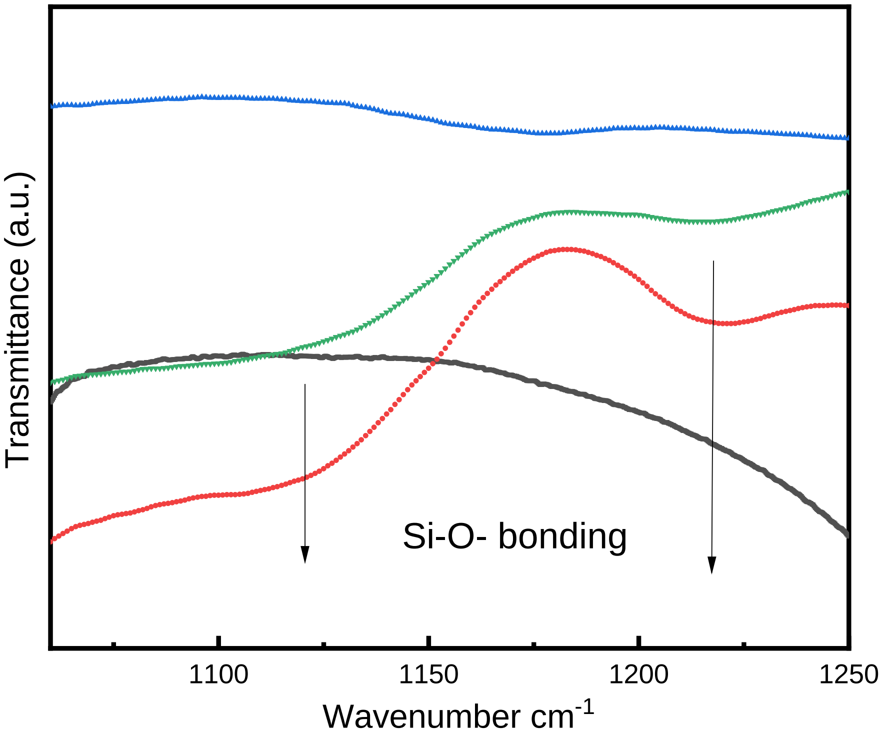

Figure S1. FTIR spectra showing Si-O stretching vibrations for the samples.

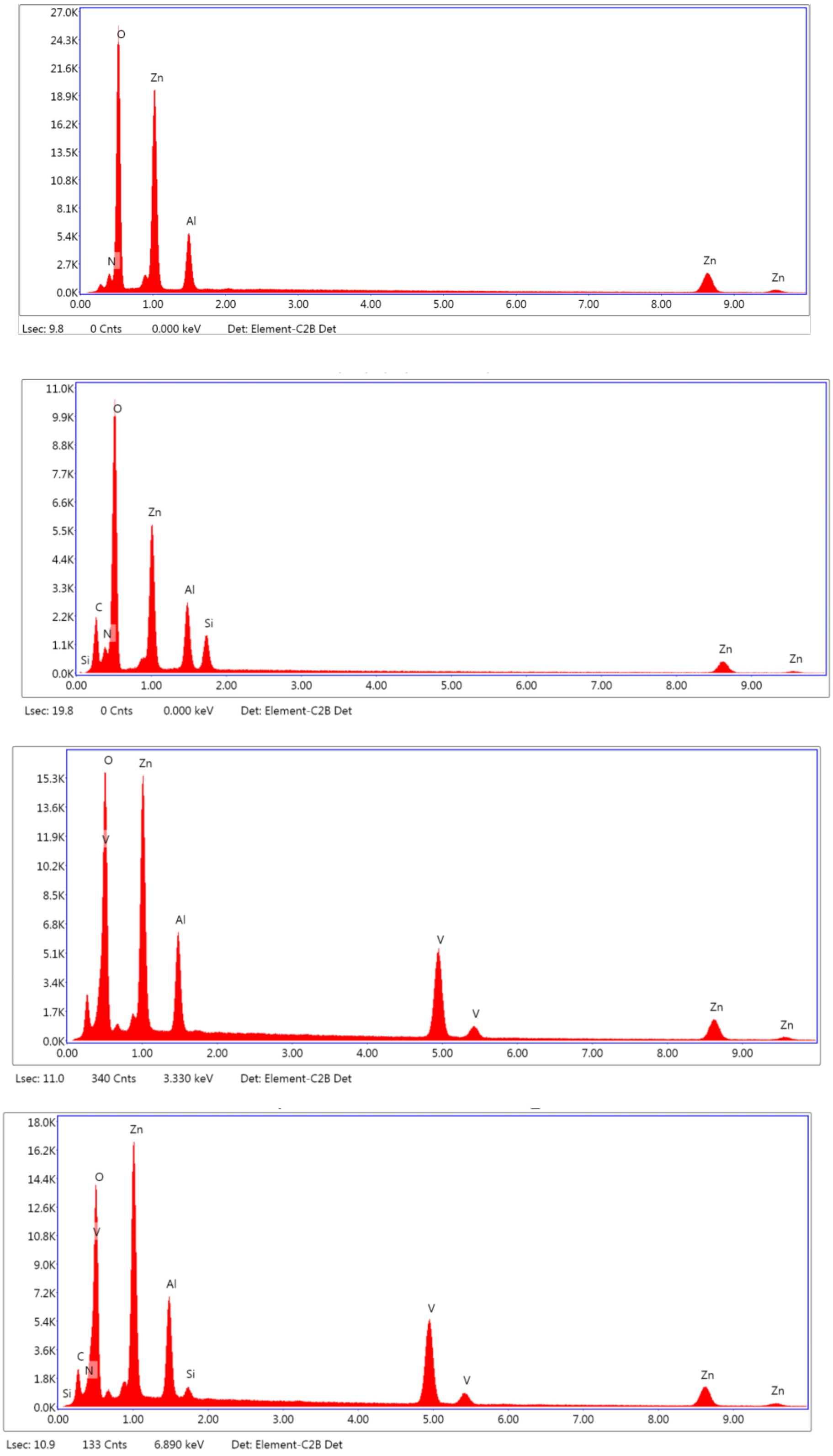

Figure S2. EDS spectra and elemental analysis of LDH-N, LDH-NF, LDH-D, and LDH-DF nanoparticles.

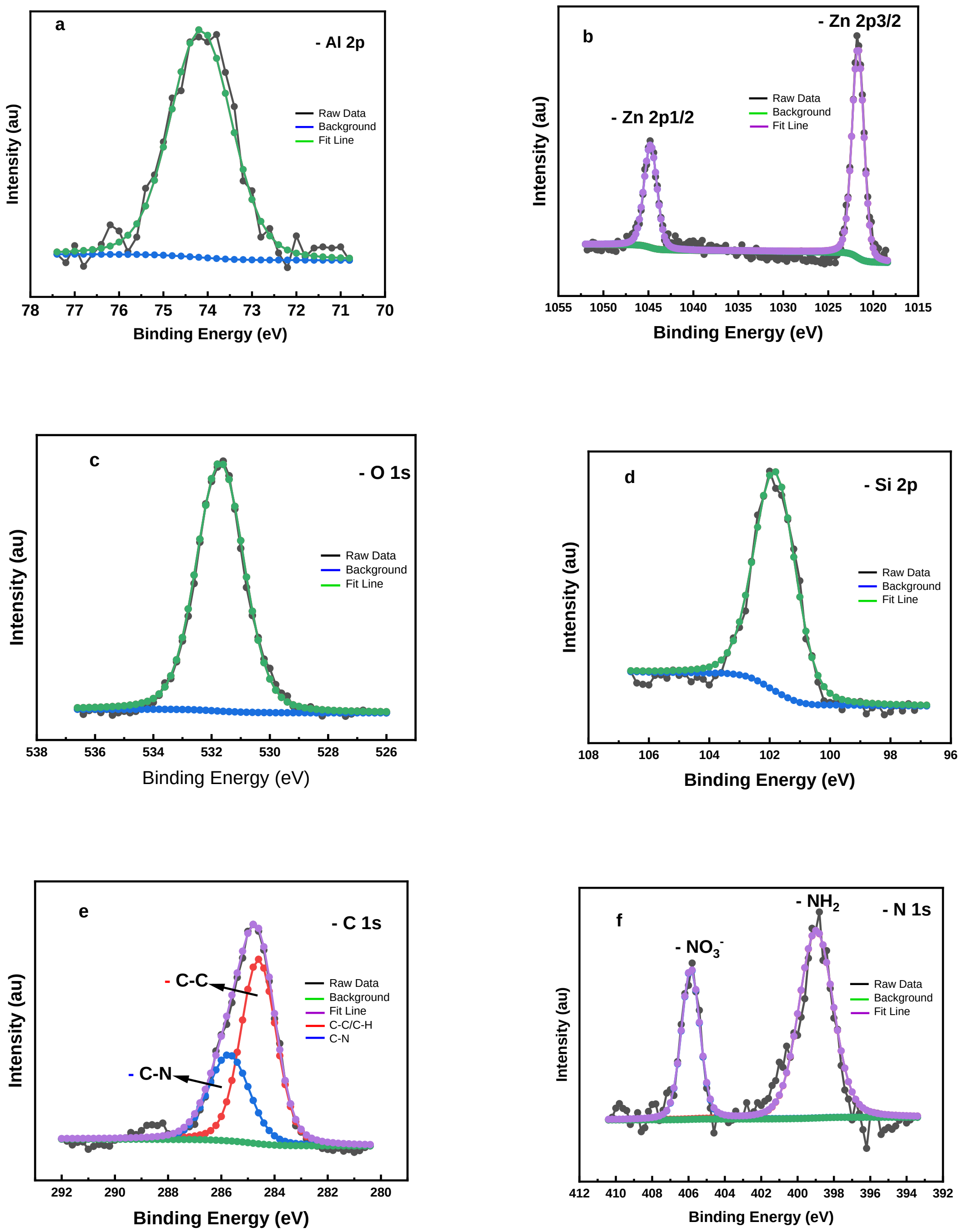

Figure S3. (a) Al 2p, (b) Zn 2p, (c) O 1s, (d) Si 2p, (e) C 1s, and (f) N 1s XPS spectra of the LDH-NF sample.

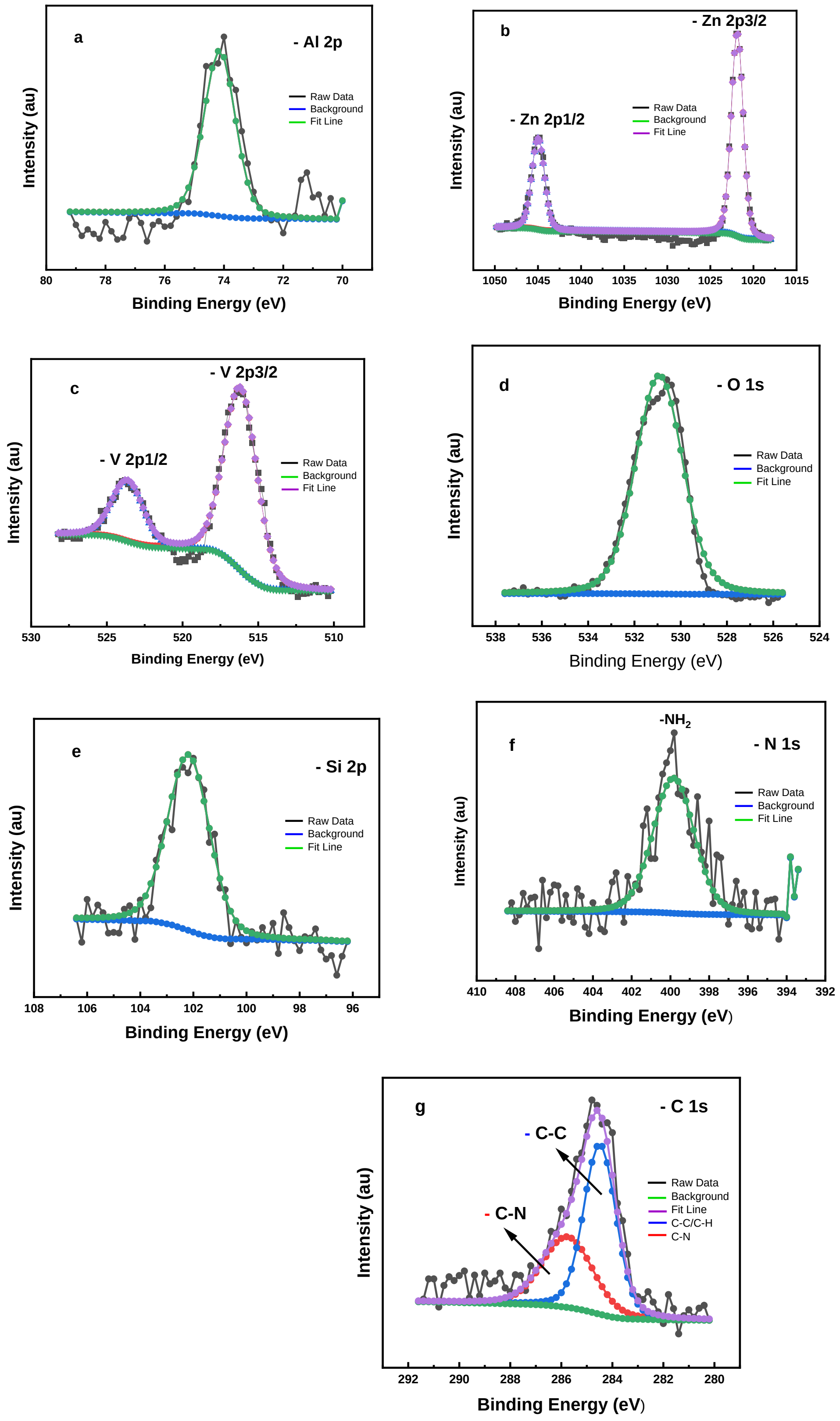

Figure S4: (a) Al 2p, (b) Zn 2p, (c) V 2p, (d) O 1s, (e) Si 2p C 1s, (f) N 1s, and (g) C 1s XPS spectra of the LDH-DF sample.

In Fig S3, Al 2p, Zn 2p, O 1s, Si 2p, C 1s and N 1s XPS spectra are shown for the LDH-NF sample. Characteristic Al and Zn spectra are seen for the LDH structure, as indicated by their main oxidation states of Al^{3+} and Zn^{2+}. The O 1s binding energy is at 531.7 eV for LDH-NF. A peak in the Si 2p spectrum for the APTES grafting can be seen at 101.8 eV, which corresponds to Si-O bonding. As can be seen, the C 1s peak is fitted with two components. The dominant peak at 284.5 eV indicating C-C bonds and the other peak at 285.7eV corresponding to C-N bond, which belongs to carbon and nitrogen atoms in aminopropyl group attached on silane structure in APTES. The N 1s spectrum also demonstrates two components at 399.9 eV and 405.8 which are attributed to NH_2 group on APTES and NO_3 interlayer anions, respectively.

Fig S4 shows measurements for Al, Zn, V, O, Si, N, and C for the LDH-DF sample. In this case, there is an extra component for LDH-DF. The V 2p core level spectrum shows two peaks for Vanadium at 523.6 eV (V 2p1/2) and 516.2 eV (V 2p3/2) related to V-O bond and providing proof of the successful anion exchange reaction and decavanadate anions present in the Zn-Al LDH structure's interlayer. There is a negative change in the O 1s binding energy from 531.69 eV for LDH-NF to 530.6 eV for LDH-DF which is most likely due to an increase in the electron density on the latter caused by the effective electronic interaction between the decavanadate anions and the Zn-Al-double hydroxide layers. In contrast to LDH-NF, LDH-DF's N 1s spectra only exhibits one component at 399.8 eV which belongs to the amine group on APTES and offers another strong proof in favor of the replacement of nitrate anions with vanadate anions. LDH-DF shows Zn2p, Al2p, Si 2p, and C 1s core levels spectra almost at the same binding energies as for LDH-NF.

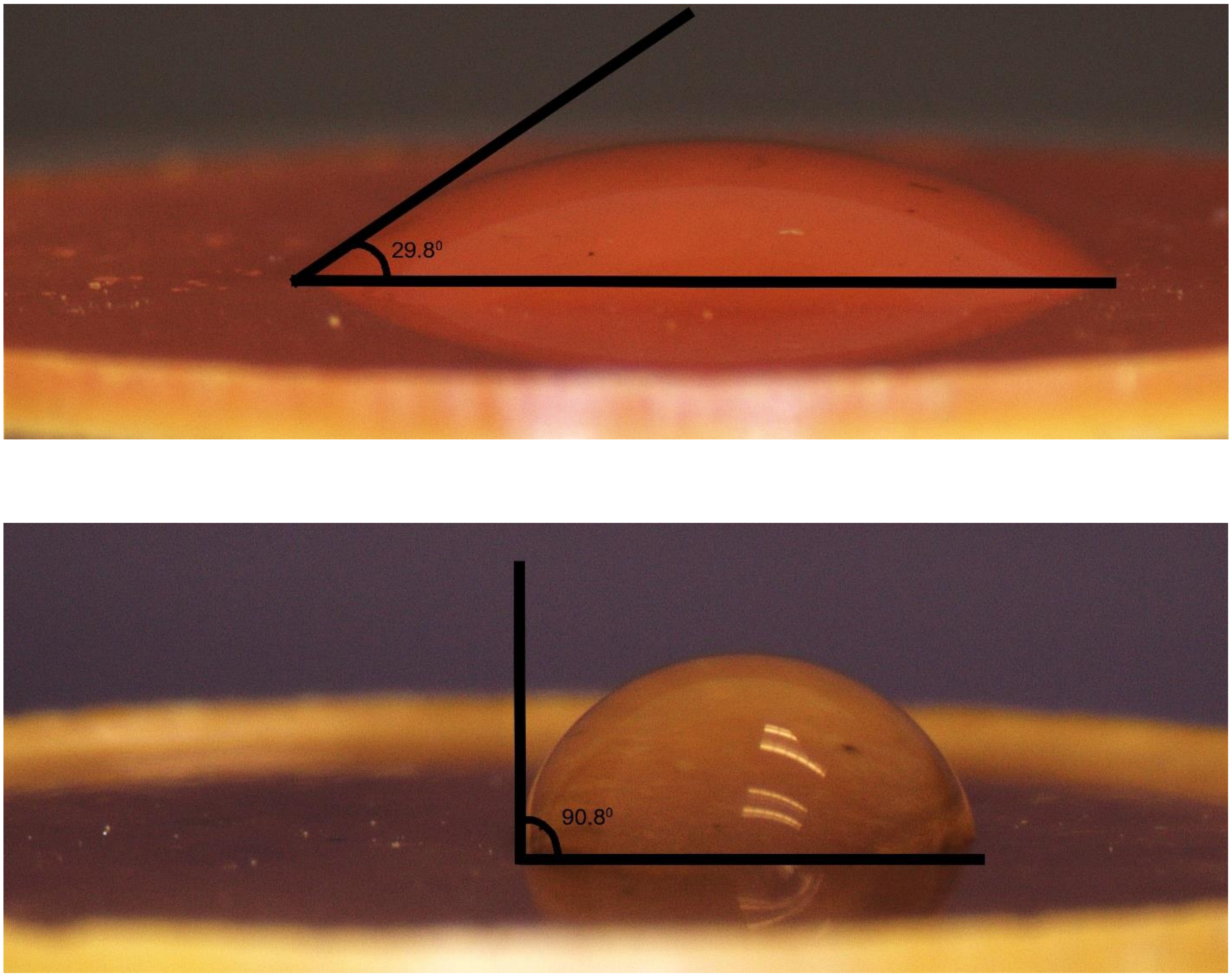

Figure S5. Contact angle measurements for LDH-D (top) and LDH-DF (bottom) pressed nanopowders.

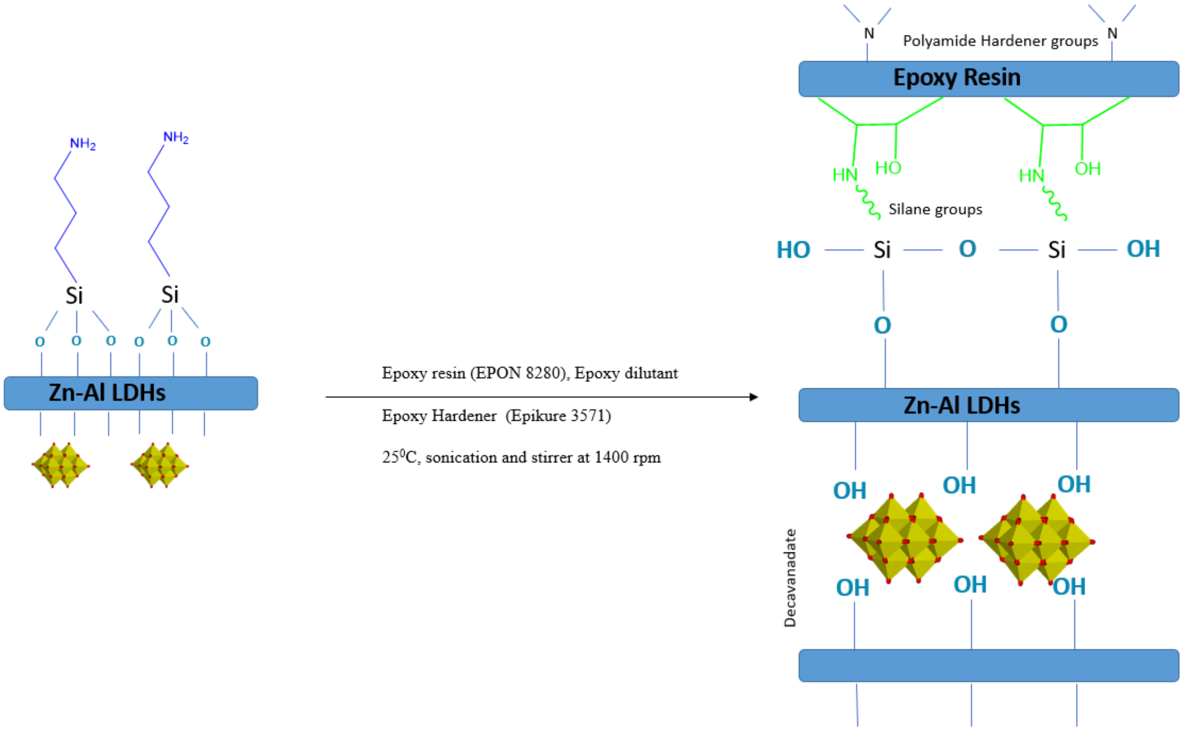

Figure S6. Schematic for the functionalization decavanadate intercalated LDH (LDH-DF) nanopowders and its interaction with epoxy matrix.
